# Variation and appropriateness of antipsychotic use in long-term care facilities across Newfoundland and Labrador

**DOI:** 10.1177/17151635211005161

**Published:** 2021-04-23

**Authors:** Zachary E. M. Giovannini-Green, John-Michael Gamble, Brendan Barrett, Zhiwei Gao, Susan Stuckless, Patrick S. Parfrey

**Affiliations:** Translational and Personalized Medicine Initiative, Memorial University of Newfoundland, St. John’s, NL; School of Pharmacy, Memorial University of Newfoundland, St. John’s, NL; School of Pharmacy, University of Waterloo, Waterloo, ON; Translational and Personalized Medicine Initiative, Memorial University of Newfoundland, St. John’s, NL; Faculty of Medicine, Memorial University of Newfoundland, St. John’s, NL; Faculty of Medicine, Memorial University of Newfoundland, St. John’s, NL; Translational and Personalized Medicine Initiative, Memorial University of Newfoundland, St. John’s, NL; Translational and Personalized Medicine Initiative, Memorial University of Newfoundland, St. John’s, NL; Faculty of Medicine, Memorial University of Newfoundland, St. John’s, NL

## Abstract

**Objective:**

The use of antipsychotics to treat seniors in long-term care facilities (LTCFs) has raised concern because of health consequences (i.e., increased risk of falls, stroke, death) in this vulnerable population. This study measured geographic patterns of antipsychotic utilization among seniors living in LTCFs in Newfoundland and Labrador (NL) and assessed potential inappropriateness.

**Method:**

We analyzed prescription records among adults 66 years and older with provincial prescription drug coverage admitted to LTCFs in NL between April 1, 2011, and March 31, 2014. Patterns of use were analyzed across the 4 regional health authorities (RHAs) in NL and LTCFs. Logistic, Poisson and linear regression models were used to test variations in prevalence, rate and volume of antipsychotic utilization. To assess potential inappropriateness of antipsychotic use, we analyzed data from Resident Assessment Instrument–Minimum Data Set (RAI-MDS) 2.0 forms from NL LTCFs between January 1, 2016, and December 31, 2018. Pearson chi-squared analysis was performed at the RHA and LTCF levels to determine changes in percentage of total prescriptions or antipsychotic prescriptions without psychosis.

**Results:**

Between 2011 and 2014, 2843 seniors were admitted to LTCFs across NL; of these, 1323 residents were prescribed 1 or more antipsychotics. Within the 3-year period, the percentage of antipsychotic use across facilities ranged from 35% to 78%. Using data from 27,260 RAI-MDS 2.0 assessments between 2016 and 2018, 71% (6995/9851) of antipsychotic prescriptions were potentially inappropriate.

**Discussion:**

There is substantial variation across NL regions concerning the utilization of antipsychotics for senior in LTCFs. Facility size and management styles may be reasons for this.

**Conclusion:**

With nearly three-quarters of antipsychotic prescriptions shown to be potentially inappropriate, systematic interventions to assess indications for antipsychotic use are warranted. *Can Pharm J (Ott)* 2021;154:xx-xx.

**Knowledge Into Practice**

- It is well known that overuse of antipsychotics is common among Canadian seniors living in long-term care facilities.
- This study found a wide variation in the prevalence of seniors in long-term care facilities being prescribed antipsychotics across the 4 regional health authorities of the province.
- The results also indicate a high (but decreasing) prevalence of potentially inappropriate antipsychotic prescription across the 4 health authorities.
- The role of pharmacists as medication experts is essential in the ongoing effort to reduce the high prevalence of potentially inappropriate antipsychotic prescribing in the province.

**Mise En Pratique Des Connaissances**

- Il est bien connu que la surconsommation d’antipsychotiques est courante chez les personnes âgées canadiennes vivant dans des établissements de soins de longue durée.
- Cette étude a révélé une grande variation dans la prévalence des personnes âgées dans les établissements de soins de longue durée qui se voient prescrire des antipsychotiques dans les quatre autorités régionales de la santé de la province.
- Les résultats indiquent également une prévalence élevée (mais décroissante) de la prescription potentiellement inappropriée d’antipsychotiques dans les quatre autorités sanitaires.
- Le rôle des pharmaciens en tant qu’experts en médication est essentiel dans l’effort continu pour réduire la prévalence élevée de la prescription potentiellement inappropriée d’antipsychotiques dans la province.

## Introduction

The high rates of potentially inappropriate antipsychotic prescriptions among seniors have been well documented in Canada and in other parts of the world.^1-3^ While approximately 5% of community-dwelling seniors receive antipsychotics,^2^ the prevalence of antipsychotic prescription in long-term care for 2016-2017 was estimated to be 21% nationally and 38% in Newfoundland and Labrador (NL).^3^ Many residents in long-term care facilities (LTCFs) are prescribed antipsychotics to treat the behavioural and psychological symptoms of dementia.^4^ This is despite Health Canada warnings regarding the risks of using atypical antipsychotics in elderly persons. Furthermore, only 1 antipsychotic agent (risperidone) is labeled for the treatment of dementia and only for the short-term management of severe behavioural problems.^5^

Studies conducted elsewhere in the world have shown substantive variation in prescribing practices for antipsychotics in LTCFs across regions.^6-9^ In Ontario, a study of residents in LTCFs found some variation between the different regions of the province in terms of antipsychotic prescriptions, ranging from 25.4% to 32.4%.^10^ A report published by the Canadian Institute for Health Information (CIHI) reported antipsychotic prescribing rates in nursing homes in 5 provinces (Prince Edward Island [PEI], New Brunswick, Manitoba, Ontario and British Columbia) from 2006 to 2014. As of 2014, Manitoba had the lowest rate of the 5 provinces, at 32%, and New Brunswick had the highest rate at over 50%.^11^ Similarly, a study conducted across multiple states in the United States found that antipsychotic prescription in LTCFs was significantly influenced by region and increased significantly as size of the facility increased.^12^

Given the high rates of antipsychotic use previously reported by the CIHI, we sought to 1) estimate the geographic variation in antipsychotic utilization across the 4 regional health authorities (RHAs) of NL and 2) report the proportion of antipsychotic use without signs of psychosis among seniors living in LTCFs in NL. Geographic variation was examined to identify potential areas in which to pursue additional study.

## Methods

### Data sources and study population

This study was composed of 2 different parts, both estimating variation in antipsychotic use. In the first part of the study, a cohort of LTCF residents in NL 66 years and older was identified who, between April 1, 2011, and March 31, 2014, received publicly funded drug coverage for eligible prescriptions, including antipsychotics, under the 65Plus Plan provided by the Newfoundland and Labrador Prescription Drug Program (NLPDP). This cohort was identified using provincially maintained health care databases linked and provided by the Newfoundland and Labrador Centre for Health Information (NLCHI).

The NLPDP was accessed to obtain prescription records for individuals receiving the Guaranteed Income Supplement (GIS) and Old Age Security (OAS) benefits. Approximately 80% of seniors in the province receive GIS due to their income being less than the federally set minimum.^13^ Information collected by the NLPDP database includes drug dispensed, date of dispensation, drug dosage, quantity dispensed and days’ supply. The Provincial Client Registry, which contains personal demographic information such as Medical Care Plan (MCP) number, sex, month and year of birth and postal code, was also used. Last, the Long-Term Care (LTC) Module of the Provincial Meditech database, containing information on seniors living in LTCFs as well as their dates of admission and discharge, was linked.

Each database contained a deidentified unique patient number to allow for linkage between databases. Only patients living in the provincially funded LTCFs were included in the study, thereby excluding the privately funded personal care homes (PCHs). Patients were also excluded from the study cohort if they spent longer than 7 consecutive days outside an LTCF, so as not to confound the data. This study has been approved by the Health Research Ethics Authority of Newfoundland and Labrador (HREB #2016.324).

For the second part of the study, antipsychotic use and appropriateness were estimated for all residents in LTCFs in NL who had quarterly Resident Assessment Instrument–Minimum Data Set (RAI-MDS) 2.0 forms completed between January 1, 2016, and December 31, 2018. The RAI-MDS 2.0 assessments are completed by a case manager (usually a nurse or a social worker) for each resident at each facility. These assessments are the standardized tool for assessing residents upon their admission to the LTCF, any significant change in health status and at the end of every calendar quarter, meaning that the progress of a single resident can be tracked over time. Data from this assessment include age, sex, month of assessment, if antipsychotics were prescribed and if the prescription was potentially inappropriate. All data for the RAI-MDS are collected in person. The initial assessment of residents upon admittance to LTCFs was not included to give facilities 1 quarter to safely discontinue any antipsychotics if required.

If a prescription for antipsychotics was noted in the RAI-MDS 2.0, the reason for the prescription was examined. Acceptable indicators for prescribing antipsychotics according to CIHI are a diagnosis of schizophrenia, Huntington chorea, delusions/hallucinations and/or the treatment of end-of-life residents.^14^ If none of these indications were noted in the assessment, the prescription was flagged as potentially inappropriate. This list is not inclusive of all possible indicators for appropriate antipsychotic prescription. Therefore, the term *potentially inappropriate* must be used to describe prescriptions for patients without indications stated above.

### Definitions of geographic regions

Seniors living in LTCFs were categorized by location in 1 of the 4 regional health authorities (RHAs) in the province: Eastern Health, Central Health, Western Health or Labrador-Grenfell Health. Of the 4 RHAs, Eastern Health is the largest by population, serving approximately 300,000 people in the eastern part of the island with 8 hospitals, 7 health centres and 15 LTCFs.^15^ Central Health serves roughly 95,000 people in the central region of the island with 2 hospitals, 9 community health centres and 11 LTCFs.^15^ Western Health serves roughly 78,000 people in western Newfoundland and most of the northern peninsula with 2 hospitals, 4 health centres and 6 LTCFs.^15^ Labrador-Grenfell Health serves roughly 37,000 people in Labrador and the top of the northern peninsula with 3 hospitals, 3 health centres and 4 LTCFs.^15^ Each LTCF in the 4 RHAs was randomly assigned a number to maintain anonymity.

### Measuring drug utilization

For the first part of the study, all residents who received 1 or more antipsychotic prescriptions during the 3-year period were defined as users. The database detailed the specific drug each senior received as well as the strength of each dose. All analyses were performed using the total number of residents receiving antipsychotic medications from 2011 to 2014. Each drug was identified by its Anatomical Therapeutic Chemical (ATC) code. Drug utilization for antipsychotic prescriptions was measured using 3 metrics: 1) prevalence of use was calculated as the ratio of total number of users per quarter for antipsychotics over total number of NLPDP beneficiaries per quarter, 2) the rate of use was calculated as the ratio of the number of antipsychotic prescription records per 1000 total prescriptions per quarter and 3) volume of use was calculated based on total number of defined daily doses (DDDs) per quarter per drug. The World Health Organization (WHO) defines DDD as the “assumed average dose per day of a drug in adults, as prescribed for its main indication.”^16^ Total DDD is calculated by obtaining the product of the dose strength and the number of days’ supply, then dividing by 1 DDD.^16^ Chronic users of antipsychotics (defined as receiving 2 or more prescriptions for 2 or more consecutive quarters) were specifically identified.

For the second part of the study, RAI-MDS 2.0 assessments were analyzed to determine overall prevalence of antipsychotic prescriptions as well as prevalence of potentially inappropriate prescriptions. Prevalence of antipsychotic use was calculated for each quarter of the years 2016 to 2018 as the ratio of assessments including a prescription for antipsychotics over the total number of assessments. The prevalence was analyzed over a 3-year period. The prevalence of potentially inappropriate prescriptions was calculated for each quarter as the ratio of assessments with no appropriate prescription for antipsychotics over the total number of assessments with prescriptions for antipsychotics. If a prescription for antipsychotics was noted in the RAI-MDS 2.0, the reason for the prescription was examined and noted to be acceptable only if 1 or more of the indicators in the CIHI criteria were present.^14^

### Statistical analysis

Descriptive statistics were used to summarize the study population characteristics across RHAs. Differences in patient characteristics between geographic regions for continuous variables were examined by *t*-test, and discrete variables were tested using chi-squared tests.

Multivariable logistic regression models were used to test regional differences in the prevalence of seniors receiving antipsychotics (dichotomous variable). Poisson regression models were used to analyze differences in the rate of prescriptions among regions. Linear regression models were used to measure differences in volume across geographic locales. The primary variable for modelling was RHA, with results indicating a *p*-value, odds ratio (or difference if linear regression) and a 95% confidence interval. In addition to comparing differences among RHAs, variation across individual facilities where at least 30 residents received antipsychotic prescriptions was measured for prevalence, rate and volume.

For the multivariate analyses, age, sex and chronic disease score (CDS) were used as covariates in the models. CDS is a prescription-based comorbidity score where 1 point is given to an individual corresponding to each drug class they are prescribed, with a maximum score of 24 points. An increased CDS is associated with increased risk of 1-year all-cause hospitalization and mortality.^17^ Age was measured as a continuous variable and sex as a binary variable (male = 0, female = 1), as it was recorded in the data set. Potential interactions between facility size and prevalence were also analyzed. Testing was completed on all models as well as the 2-way interactions for the 3 variables used in the models. No violations of the assumptions for the 3 regression models were found.

For the second study, a Pearson chi-squared analysis was performed on the provincial as well as individual RHA results to determine any statistically significant changes in prevalence of total or potentially inappropriate antipsychotic prescriptions.

All statistical analyses were conducted using SPSS, version 23 (SPSS, Inc., Chicago, IL).

## Results

### Long-term care facilities in the province by region

Using the prescription claims-based cohort, we identified a total of 2843 residents in the LTCFs across the 4 RHAs, 1323 of whom received prescriptions for antipsychotics (Table 1). Of those residents receiving antipsychotics, 1134 (86%) were chronic users. There were no noticeable differences in the patterns of use for short-term and chronic users of antipsychotics. Of the 21,495 antipsychotic prescriptions written in the 3-year period, nearly all were for a 1-month period. The prevalence, rate and volume of antipsychotic prescriptions in the RHAs were highest in Eastern Health and lowest in Labrador-Grenfell Health and remained relatively stable over the 3-year period from 2011 to 2014 (Table 1). The median age of the residents in each of the RHAs was between 83 and 86 years, and the prevalence of males in long-term care was between 23% and 35%. The median CDS was 5.00 in Eastern Health, Central Health and Western Health and 4.50 in Labrador-Grenfell Health. None of the differences in demographic information was statistically significant. After controlling for age, sex and CDS, there was no significant difference in the prevalence, rate or volume between Eastern Health and the other RHAs (*p* = 0.120, *p* = 0.125 and *p* = 0.088, respectively).

**Table 1:** Prevalence, rate and volume of antipsychotic prescriptions in the regional health authorities from 2011 to 2014

Characteristic	Eastern Health	Central Health	Western Health	Labrador-Grenfell Health
Residents in long-term care facilities	1983	659	153	48
Prevalence of residents receiving antipsychotics, *n* (% residents)	952 (48%)	285 (43%)	69 (45%)	17 (35%)
Rate of antipsychotic prescriptions per 1000 total	58	47	50	50
Volume of antipsychotic prescriptions as defined daily doses/1000 people/day	95	65	62	52

Province-wide, 27,260 RAI-MDS 2.0 assessments were analyzed over a 3-year period from 2016 to 2018 in the 35 provincially funded LTCFs. Out of the total number of assessments, 8718 were completed in 2016, 9190 in 2017 and 9352 in 2018. During these 3 years, 3377 (39%) assessments in 2016, 3398 (37%) assessments in 2017 and 3076 (33%) assessments in 2018 showed a prescription for antipsychotics. Of the assessments showing a prescription for antipsychotics, 2467 (73%) assessments in 2016, 2498 (74%) assessments in 2017 and 2030 (66%) assessments in 2018 were deemed to be potentially inappropriate. The decrease in the total number of assessments with antipsychotics prescribed between 2016 and 2018 was shown to be statistically significant by a Pearson chi-squared test (*p* < 0.05). The decrease in the number of assessments with potentially inappropriate antipsychotic prescriptions between 2016 and 2018 was also statistically significant.

### Variation in antipsychotic utilization across LTCFs

**Figure 1:**
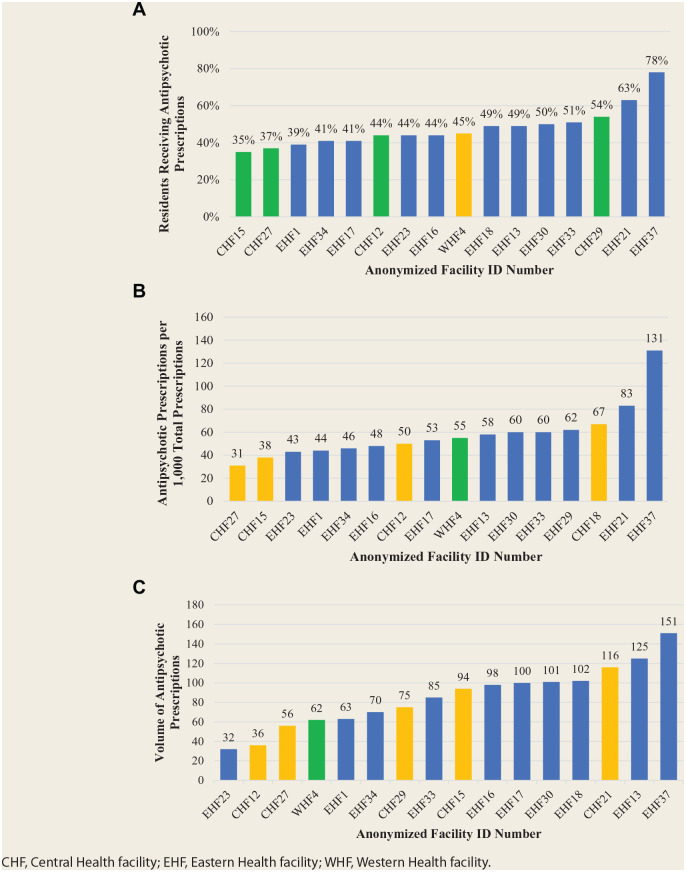
(A) Prevalence of residents in long-term care receiving one or more antipsychotic prescriptions by facility. (B) Rate of antipsychotic prescriptions filled in long-term care per 1000 total prescriptions by facility. (C) Volume of antipsychotic prescriptions filled in long-term care by facility measured as defined daily doses per 1000 people per day

Using the claims-based cohort and controlling for age, sex and CDS, the difference in prevalence between facilities for antipsychotic prescription was significant (*p* < 0.001), with a 2-fold relative variation (Figure 1A). There was no interaction between facility and age, sex or CDS. After controlling for age, sex and CDS, neither rate nor volume of antipsychotic prescriptions was statistically significant across the facilities (*p* = 0.248 and *p* = 0.087, respectively) (Figure 1B,C). Since no facilities in the Labrador-Grenfell region had 30 or more residents receiving antipsychotics, the region was not included in the attached figures.

**Figure 2:**
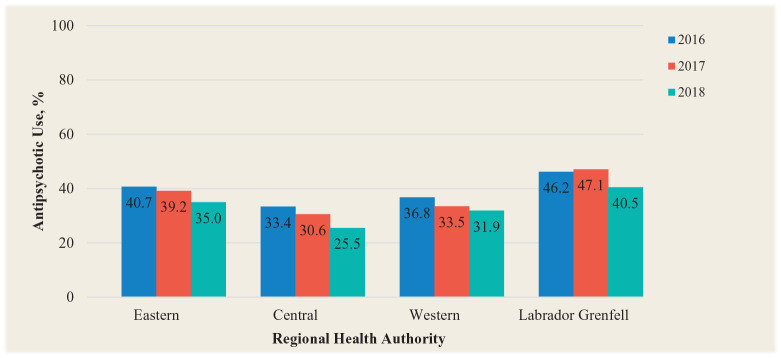
Percentage of residents receiving antipsychotic prescriptions by regional health authority based on Resident Assessment Instrument–Minimum Data Set assessments

**Figure 3:**
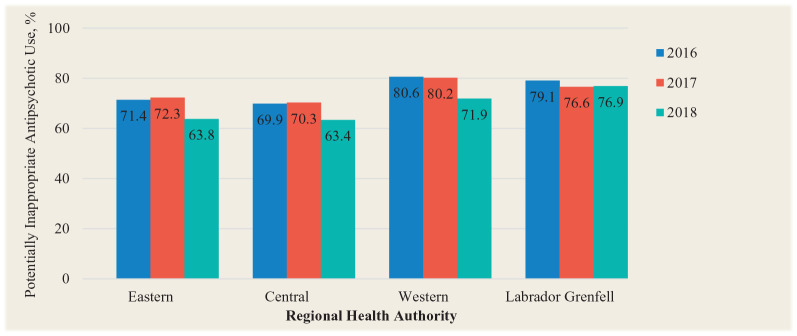
Percentage of antipsychotic prescriptions that were potentially inappropriate by regional health authority based on Resident Assessment Instrument–Minimum Data Set assessments

For the second part of the study, the prevalence of antipsychotic use in Eastern Health LTCFs ranged from 14.7% to 80.0% over the 3-year period, with a mean of 40.7% in 2016, 39.2% in 2017 and 35.0% in 2018 (Figure 2). The percentage of potentially inappropriate antipsychotic prescriptions ranged from 38.9% to 100.0% over the 2-year period, with a mean of 71.4% in 2016, 72.3% in 2017 and 63.8% in 2018 (Figure 3). The prevalence of antipsychotic use in Central Health LTCFs over the 3-year period ranged from 8.9% to 63.9%, and the percentage of potentially inappropriate antipsychotic prescriptions ranged from 7.1% to 100.0%. The prevalence of antipsychotic use in Western Health LTCFs over the 3-year period ranged from 10.4% to 49.4%. The percentage of potentially inappropriate antipsychotic prescriptions ranged from 33.3% to 100.0%. The prevalence of antipsychotic use in Labrador-Grenfell Health LTCFs over the 3-year period ranged from 16.7% to 64.0%, and the prevalence of potentially inappropriate antipsychotic prescriptions ranged from 58.9% to 100.0%.

## Discussion

Our findings in both parts of the study suggest substantial variation exists across LTCFs for the use of antipsychotic medications. These findings also indicate the high level of potentially inappropriate use of antipsychotics in LTCFs in the province. However, some consolation can be found in the fact that these prevalences are decreasing over time in all 4 RHAs.

One factor that may contribute to the difference in prevalence of antipsychotic prescriptions is facility size. As shown in the study by Chen et al.,^12^ facility size is significantly and positively correlated to prevalence of psychoactive drug prescriptions. Eastern Health houses 70% of facility-dwelling seniors and administers 9 of the 10 largest facilities in the province. Study results show that the region also has the highest prevalence of antipsychotic prescription.

In contrast to the first study, LTCFs in Labrador-Grenfell Health had the highest prevalence of antipsychotic prescriptions between 2016 and 2018. A possible reason for this is the difference in the information collected in the 2 studies, with study 1 collecting data from residents 66 years or older and on the NLPDP. Study 2, on the other hand, collected information on all LTCF residents.

A more intangible factor to explain differences in utilization patterns is the work culture of the RHAs and their individual facilities. Hughes et al.^18^ wrote that the individual culture of a facility greatly influences the running of the facility, including which residents do or do not receive prescriptions for potentially harmful medications. Various agencies in Canada have addressed ways to change facility culture to implement healthier prescribing practices. These include agreeing on appropriateness criteria, establishing regular reviews and pursuing nonpharmacologic interventions.^19,20^

In 2015, the Western and Central Health RHAs initiated a deprescribing program in their LTCFs in conjunction with the Canadian Foundation for Health Care Improvement (CFHI).^21^ The program was aimed specifically at discontinuing inappropriate antipsychotic prescriptions within LTCFs. Nationally, 2 studies released by the Canadian Institute for Health Information^11,22^ have indicated that total prevalence of antipsychotic prescriptions for seniors has decreased in some provinces (e.g., Ontario and Manitoba) but increased in others (e.g., all Atlantic provinces). The high median age of the population in the Atlantic provinces may be contributing to this phenomenon,^23^ as well as more developed policies being put in place by other provinces.^20^

Solutions are not always simple, however. Behavioural interventions often require a high staff-to-resident ratio to personalize the health care management of residents. A lack of human resources means that staff working in LTCFs may have few alternatives to the prescription of antipsychotics to treat behavioural symptoms in residents. Interventions may, however, be available from outside the LTCFs. The Medication Therapy Services (MTS) clinic operated by the School of Pharmacy at Memorial University offers services to patients in the community, their doctors and other health providers, to help them receive the medications that are best for them.^24^ Starting in 2017, a pilot study was launched by the MTS clinic to provide deprescribing services to residents in LTCFs.^25^

### Limitations

The first part of the study included residents covered under NLPDP and living in government-funded LTCFs. As well as excluding all residents in private facilities, the study also excludes seniors ineligible for prescription drug coverage under NLPDP. Approximately 80% of seniors in Newfoundland and Labrador receive coverage from this program.^13^ Since the Western and Labrador-Grenfell Health regions had relatively fewer residents (together constituting only 7% of the total number of residents), some results from the regression analyses may have less precision than the results from the Eastern and Central RHAs. In addition, all LTCFs were included in this analysis, even those where there were very few residents receiving antipsychotic prescriptions. While this information is important in order to understand the utilization of antipsychotics in all facilities in the province, the results may again not be as precise as in larger facilities. Finally, there were seniors who had to be removed from both parts of the study because their relevant data were missing or could not be used. It is unlikely that those excluded from the study had any real effect, as the number of those excluded was less than 2% of the total and their demographic characteristics were similar to those included in the study.

For the second study, in keeping with CIHI guidelines,^14^ the use of antipsychotics was deemed appropriate only for end-of-life residents and residents with psychosis. This excludes the accepted short-term use of antipsychotics to manage inappropriate behaviours, such as aggression,^5^ in residents. Limitations that affect both studies include no clinical indication for the prescription of antipsychotics, no data on the specialty of the prescribing physician and no data on the clinical outcomes of the resident being treated with antipsychotics to monitor effectiveness or tolerability.

## Conclusions

These analyses were designed to measure geographic variation of antipsychotic prescriptions among residents in LTCFs in Newfoundland and Labrador. It was found that the prevalence of antipsychotic prescriptions was significantly different across geographic regions in LTCFs but also has been decreasing in recent years in each of the 4 RHAs. The prevalence of the potentially inappropriate prescription of antipsychotics has similarly decreased in all RHAs during the 3-year period of measurement from 2016 to 2018.
